# 1782. Antimicrobial Stewardship Opportunities in Gram-Negative Bacteremia Treatment at a Community Teaching Hospital

**DOI:** 10.1093/ofid/ofac492.1412

**Published:** 2022-12-15

**Authors:** Matthew Ficinski, Maryrose R Laguio-Vila

**Affiliations:** Rochester General Hospital, Rochester, NY; Rochester Regional Health, Rochester, New York

## Abstract

**Background:**

Gram Negative Bacteremia (GNB) is frequently encountered among hospitalized patients. In contrast to traditional 14-day lengths of treatment (LOT), recent literature supports a shorter (7-day) LOT for uncomplicated GNB with adequate source control, and the effectiveness of oral antibiotics. The goal of the following is to outline current practices of GNB treatment and identify opportunities for antibiotic stewardship (AS).

**Methods:**

This study retrospectively reviewed all cases of uncomplicated GNB at a 528-bed community teaching hospital in Rochester, NY from January 2021 through March 2022. Demographic, laboratory, microbiologic, antibiotic therapy data, results of follow-up blood cultures (FUBC), hospital length of stay and 30-day readmission were collected. Exclusions were complicated or polymicrobial bacteremia, deaths during treatment, or prolonged hospitalization due to other medical factors. Influences of Infectious diseases (ID) or AS consult on treatment and outcomes were compared to cases with no consult. Continuous variables were analyzed using unpaired t-tests; categorical variables were analyzed using Fischer’s exact test and Chi-square as appropriate.

**Results:**

133 cases met inclusion criteria. Demographic and laboratory data are in Table 1. The frequency of bacteria isolated and source of infection are in Figure 1 and Figure 2. ID was significantly more often consulted for central line infections (17% vs 3%, p=0.01), and significantly less frequently involved in urinary tract infections (39% vs 69%, p=0.009). While total LOT were similarly longer than current literature supports (11.7 vs 12.5 days, p=0.2644), cases without ID consultation received significantly more days of oral treatment (4.7 vs 7.1 days, p=0.0275). There were no significant differences between receipt and no receipt of AS recommendations.

**Figure:**
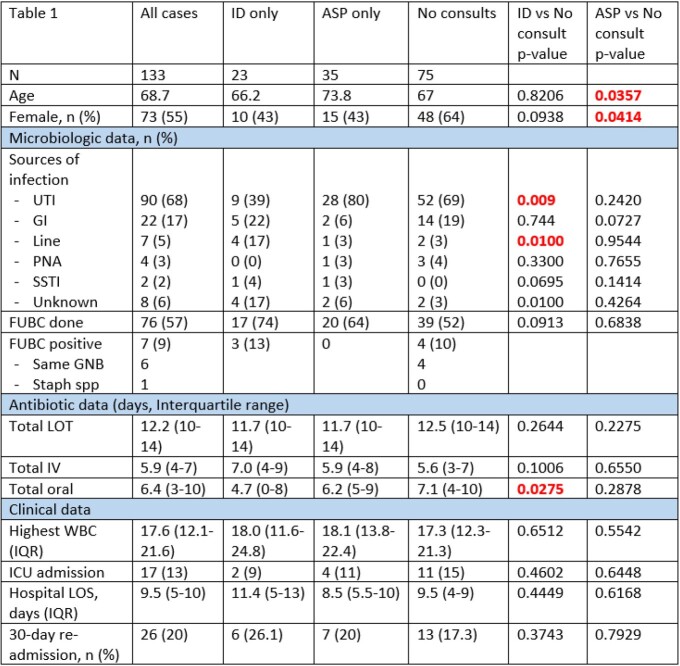

**Figure:**
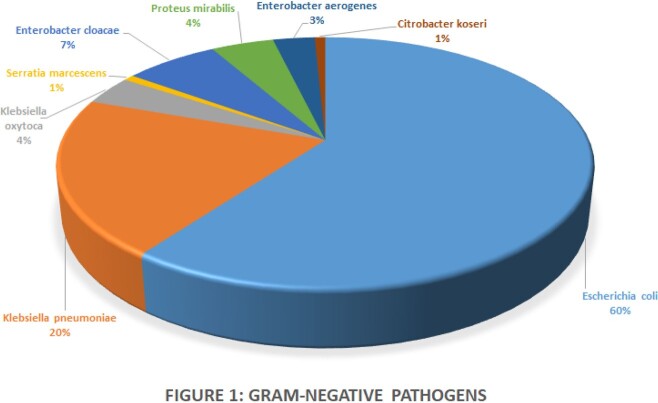

**Figure:**
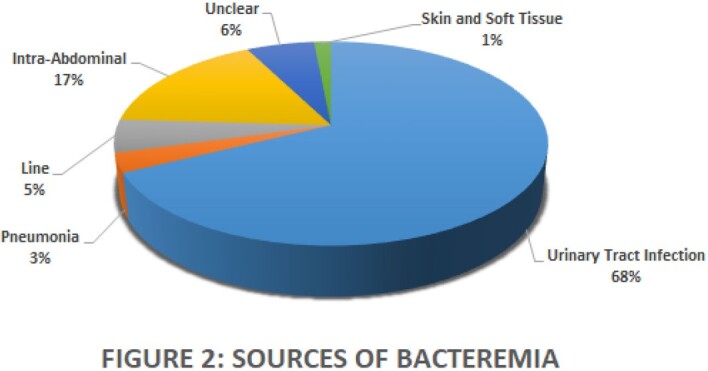

**Conclusion:**

GNB continued to receive longer LOTs than current literature recommends, with longer IV durations recommended by ID consultants compared to those without ID consult. Educational initiatives regarding the safety of shorter LOT for GNB, including the efficacy of oral antibiotics, are needed and should include ID specialists.

**Disclosures:**

**All Authors**: No reported disclosures.

